# Supplementary material for: Insulin signaling regulates R2 retrotransposon expression to orchestrate transgenerational rDNA copy number maintenance
Source: Nat Commun. 2025 Jan 4;16:399. doi: 10.1038/s41467-024-55725-6 (PMC11700107; doi:10.1038/s41467-024-55725-6)
Supplement: Supplementary file 1 — Supplementary Information [file 41467_2024_55725_MOESM1_ESM.pdf]

Supplementary Information

Supplementary Figures 1-6

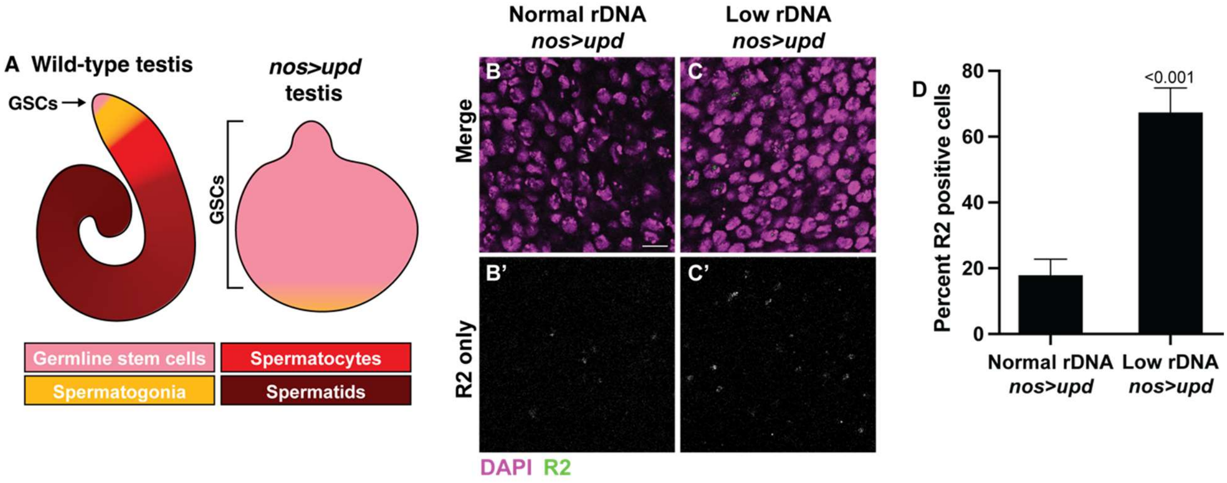

Supplementary Figure 1. R2 is regulated by rDNA CN in *nos>upd* overproliferative GSCs. A Diagram of proportion of germ cell types in in wild-type and *upd* over-expression testes. **B-C** Images of R2 RNA FISH in *nos>upd* testes in normal and low rDNA conditions. R2 in green, DAPI in magenta. GSCs identified by round nuclear morphology. Scale bar = 10 μm. **B'-C'** R2 channel only. **D** Percent R2 positive GSCs in *nos>upd* tested. Error = 95% CI. P-value determined by two-tailed chi-squared test. Normal rDNA sample (control) n = 240, Low rDNA sample n = 150. Genotypes: bb^{Z9} / Ybb^{+} ; *UAS-upd / + ; nos-Gal4 /* + (Normal rDNA *nos>upd*) and bb^{Z9} / Ybb^{0} ; *UAS-upd / +; nos-Gal4 /* + (Low rDNA *nos>upd)*. Source data are provided as a Source Data file.

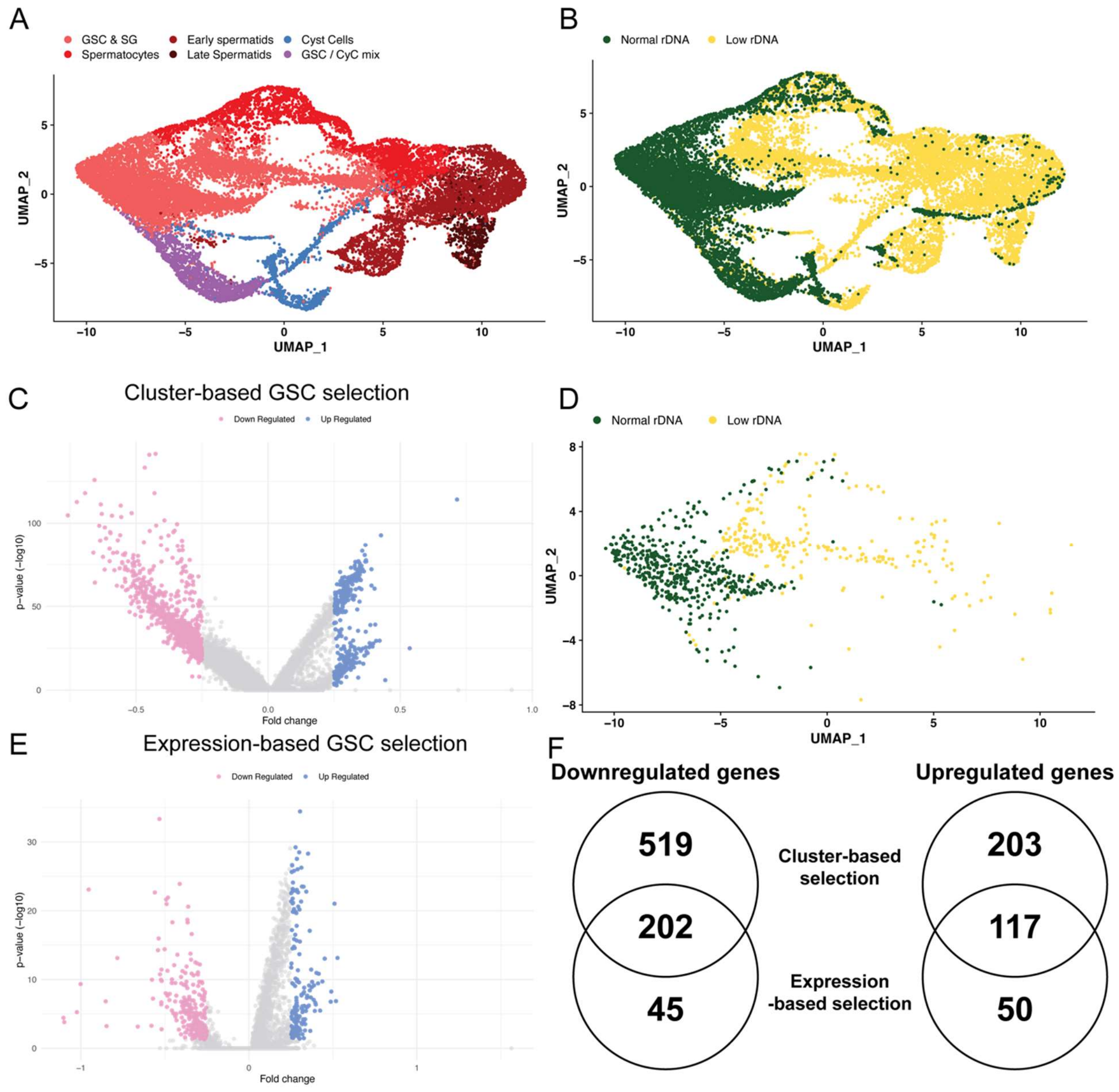

Supplementary Figure 2. Single cell sequencing analysis of *nos>upd* testes. A-B UMAP plot from all cells from combined analysis of low and normal rDNA CN *nos>upd* testes with assigned cell type **(A)** or rDNA CN condition **(B)**. **C** Differential gene expression of GSCs determined by sub-clustering. **D** UMAP plot of GSCs selected for differential gene expression based on expression of specific GSC markers. **E** Differential gene expression of GSCs determined by expression profile. **F** Overlap of up- and downregulated genes between low and normal rDNA CN GSCs selected by cluster or expression-based methods. Differentially

expressed genes determined by an absolute Log_2 Fold Change >0.25 and an adjusted p-value <0.05. Source data are provided as a Source Data file.

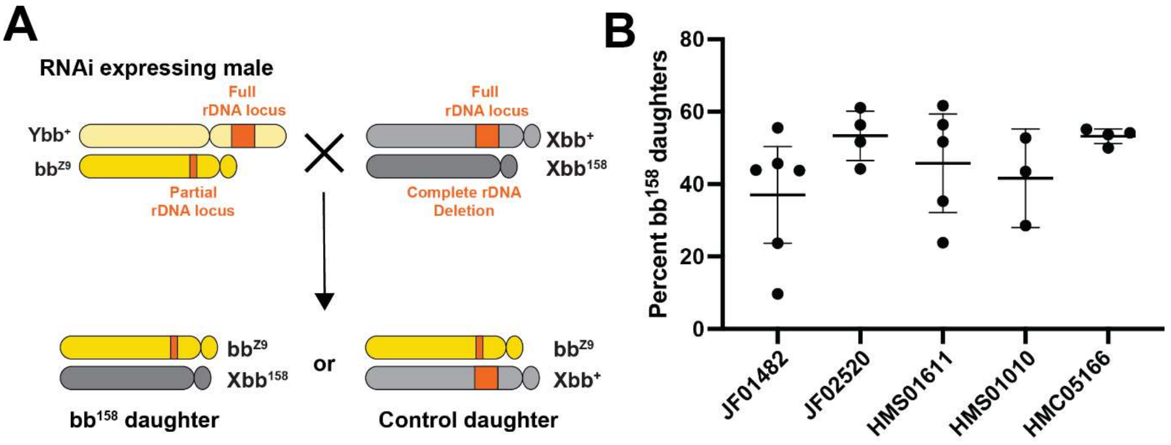

Supplementary Figure 3. rDNA magnification activity does not affect offspring viability. A Scheme to assess viability of *bb*^{Z9} / *bb*^{158} offspring from RNAi expressing males. Males harboring the partially rDNA deficient *bb*^{Z9} X chromosome and expressing RNAi targeting candidate rDNA magnification regulators were mated to females heterozygous for the completely rDNA deficient *bb*^{158} X chromosome and an X chromosome with a complete rDNA locus (*Xbb*$^{+}$). Resultant daughters all inherit the *bb*^{Z9} chromosome from their fathers, and either the *bb*^{158} or *Xbb*$^{+}$ chromosome from their mother. Therefore, it is expected that 50% of all daughters should have the *bb*^{158} chromosome, and any reduced viability *bb*^{Z9} / *bb*^{158} daughters would result in a significantly lower percentage *bb*^{158} daughters. **B** Percent *bb*^{158} daughters from a subset of candidate rDNA regulator RNAi lines tested. Each data point represents the percent *bb*^{158} daughters from an individual cross. There is no difference significant difference in percent *bb*^{158} daughters between the lines tested based on One-way ANOVA (p = 0.2729) and Tukey's multiple comparison analysis (lowest p value = 0.3376 between TRiP.JF01482 and TRiP.JF02520). RNAi lines tested are: TRiP.JF01482 (n = 6) – target gene InR and 34.45% magnified; TRiP.JF02520 (n = 4) – RpL10, 28.57% magnified; TRiP.HMS01611 (n = 5) – Atg17, 5.66% magnified; TRiP.HMS01010 (n = 3) – ankirin, 2.78% magnified; TRiP.HMC05166 (n = 4) - TotC, 0.54% magnified (see Supplementary Data 3). Source data are provided as a Source Data file.

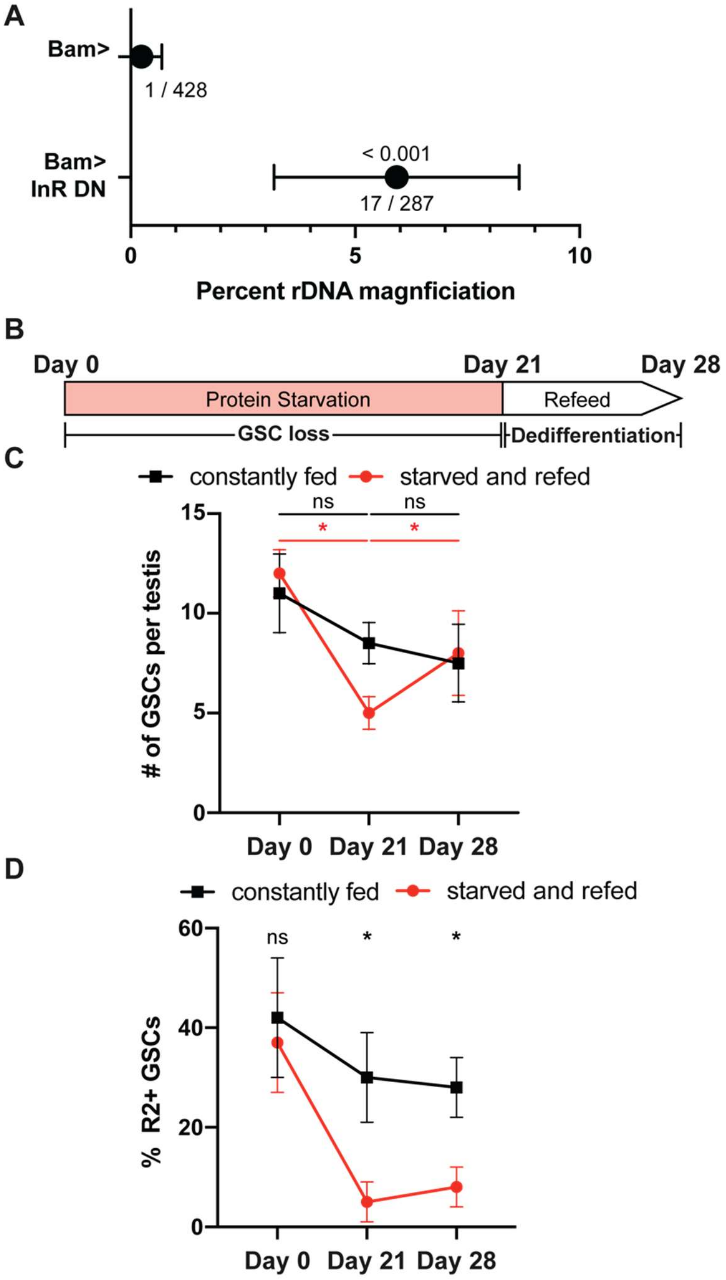

Supplementary Figure 4. InR activity suppresses rDNA magnification in SGs. A Percent rDNA magnification from males with normal rDNA CN expressing dominant negative *InR*^{K1409A} allele in SG. P-value determined by chi-squared test. Error: 95% CI. **B** Protein starvation and refeeding paradigm to induce SG dedifferentiation into GSCs. GSC loss occurs during complete protein starvation over 21 days. Refeeding on standard food promotes SG dedifferentiation into

GSCs. **C** Mean GSCs per testis in low rDNA CN animals in protein starvation dedifferentiation paradigm. Statistical significance determined between time points for control (constantly fed) or dedifferentiation (starved and refed) conditions determined by one-way ANOVA followed by Tukey's multiple comparison analysis. N = 15 for all samples. * indicates $p < 0.01$. Error: 95% CI. Exact p-values: Day 0 fed to Day 21 fed = 0.060; Day 21 fed to Day 28 fed = 0.281; Day 0 starved to Day 21 starved = $1.39 * 10^{-8}$; Day 21 starved to Day 28 starved = $2.93 * 10^{-3}$. **D** Percentage of GSCs expressing R2. Statistical significance determined between control (constantly fed) and dedifferentiation (starved and refed) conditions at each time point determined by two-tailed chi-squared test. * indicates $p < 0.01$. Error: 95% CI. 0-day timepoint p = 0.628 and n = 85 for control and 75 for dedifferentiation condition; 21-day timepoint p = 6.46×10^{-6} and n = 99 for control and 105 for dedifferentiation condition; 28-day timepoint p = 3.35×10^{-5} and n = 150 for control and 128 for dedifferentiation condition. Genotypes: *bb^{Z9} / Ybb^{+} ; UAS- InR^{K1409A} /+ ; nos-Gal4 / UAS-R2 RNAi-1* (InR DN + R2 RNAi), *bb^{Z9} / Ybb^{+} ; UAS-InR^{K1409A} /+ ; nos-Gal4 / UAS-GFP* (Normal rDNA + GFP), *bb^{Z9} / Ybb^{+} ; ; Bam-Gal4 /* + (Bam>), and *bb^{Z9} / Ybb^{+} ; ; Bam-Gal4 / UAS-InR^{K1409A}* (Bam>InR DN). All samples for C-D are *bb^{Z9} / Ybb^{0}*. Source data are provided as a Source Data file.

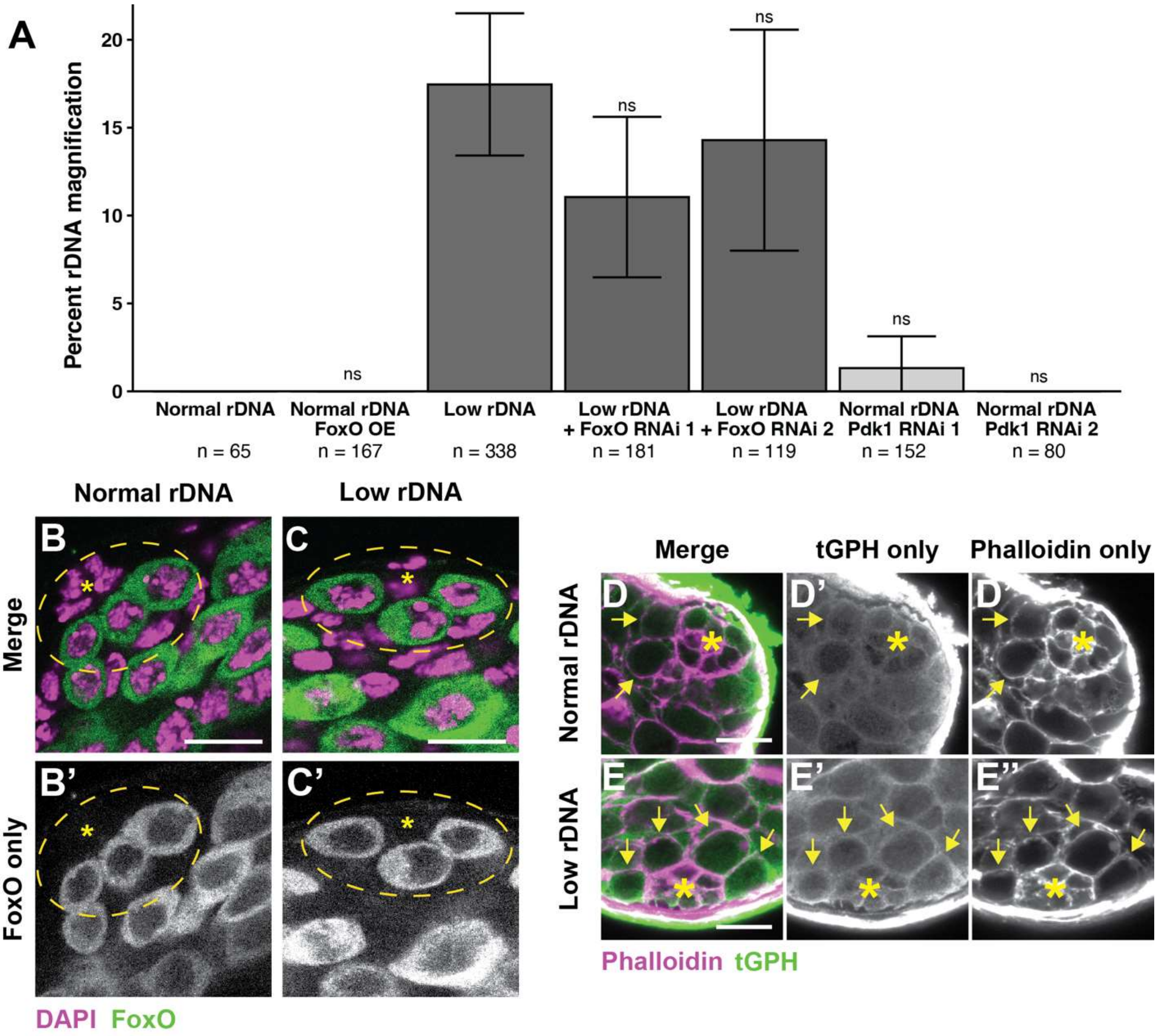

Supplementary Figure 5. Pi3K signaling does not regulate rDNA CN expansion in GSCs. **A** Percent offspring with wild-type cuticles in rDNA magnification assays from animals with manipulated Pi3K pathway factors. P-value determined by two-tailed chi-squared compared to non-transgene condition of same rDNA content (Normal rDNA is control for Normal rDNA FoxO OE, Normal rDNA PdK1 RNAi 1 and Normal rDNA Pdk1 RNAi 2 samples; Low rDNA is control for Low rDNA + FoxO RNAi 1 and Low rDNA + FoxO RNAi 2 samples). Error = 95% CI. Exact p-values: Normal rDNA FoxO OE = 1.0; Low rDNA + FoxO RNAi 1 = 0.141; Low rDNA + FoxO RNAi 2 = 1.0; Normal rDNA PdK1 RNAi 1 = 1.0; Normal rDNA PdK1 RNAi 2 = 1.0. **B-C** FoxO localization in animals expressing FoxO GFP in germ cells with low and normal rDNA CN.

Asterisk indicates hub. GSCs in dotted yellow circle. DAPI in magenta, and FoxO GFP in Green. Scale bar = 10 µm. Two replicate experiments were used to confirm results. **D-E** Pi3 phosphorylation indicated by tGPH membrane localization (Green) in animals with normal and low rDNA CN. Cell membrane marked with phalloidin (Magenta). Asterisk indicates hub. GSCs indicated by yellow arrows. Scale bar = 10 µm. Two replicate experiments were used to confirm results. Genotypes: *bb^{Z9} / Ybb^{+} ;; nos-Gal4 /* + (Normal rDNA), *bb^{Z9} / Ybb^{+} ; UAS-FoxO-GFP /* + *; nos-Gal4 /* + (Normal rDNA FoxO OE), *bb^{Z9} / Ybb^{0} ; ; nos-Gal4 /* + (Low rDNA), *bb^{Z9} / Ybb^{0} ; ; nos-Gal4 / FoxO $RNAi^{JF2019}$* (Low rDNA + FoxO RNAi 1), *bb^{Z9} / Ybb^{0} ; ; nos-Gal4 / FoxO $RNAi^{JF02739}$* (Low rDNA + FoxO RNAi 2), *bb^{Z9} / Ybb^{+} ; ; nos-Gal4 / Pdk1 $RNAi^{GL00489}$* (Normal rDNA + Pdk1 RNAi 1), *bb^{Z9} / Ybb^{+} ; ; nos-Gal4 / Pdk1 $RNAi^{GL00489}$* (Normal rDNA + Pdk1 RNAi 1), *nos-Gal4 / Pdk1 $RNAi^{HMS01250}$* (Normal rDNA + Pdk1 RNAi 2). Source data are provided as a Source Data file.

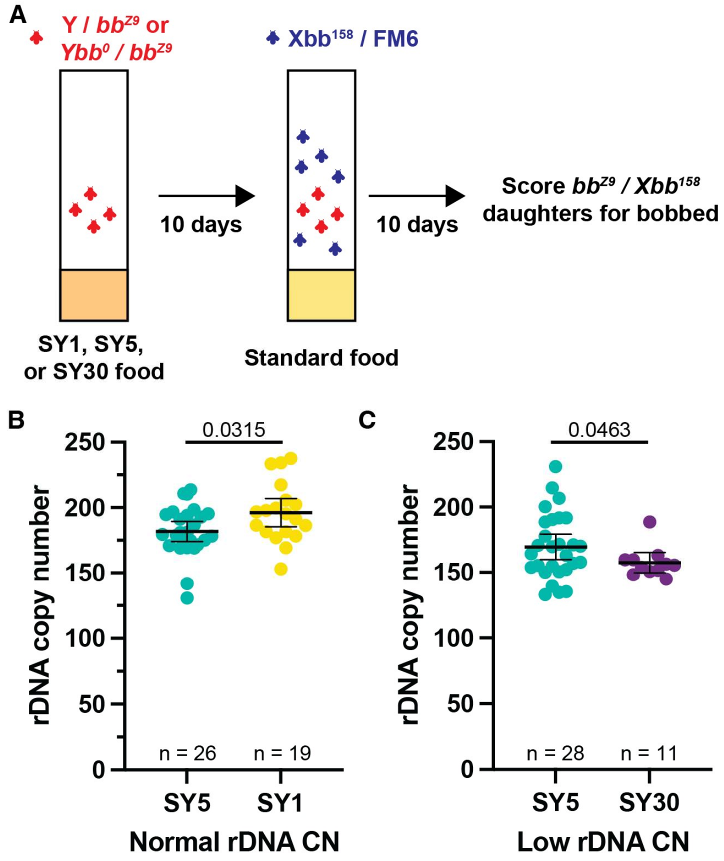

Supplementary Figure 6. Dietary effects on inherited rDNA copy number. A Schematic of testing dietary impact on rDNA magnification. Low rDNA CN (*Ybb*^{0} / *bb*^{Z9}) males were fed on SY5 or SY30 food and normal rDNA CN (Y / *bb*^{Z9}) males were fed on SY1 or SY5 food for their first 10 days of adulthood. 10 – 15 males were used per vial. Males were then mated in bulk on standard food (8 males and 16 females) to *Xbb*^{158} / *FM6* females for 10 days. Resulting *bb*^{Z9} / *Xbb*^{158} female offspring were scored for presence of bobbed phenotype. **B** rDNA copy number in offspring of fathers with normal rDNA CN fed normal (SY5) or low calorie (SY1) diet, determined by ddPCR. **C** rDNA copy number of fathers with low rDNA CN fed normal (SY5) or high calorie (SY30) diet. P-value determined by Welch's t-test. Error: 95% CI. Source data are provided as a Source Data file.
