# Supplementary material for: Insulin signaling regulates R2 retrotransposon expression to orchestrate transgenerational rDNA copy number maintenance
Source: Nat Commun. 2025 Jan 4;16:399. doi: 10.1038/s41467-024-55725-6 (PMC11700107; doi:10.1038/s41467-024-55725-6)
Supplement: Supplementary file 2 — Description of Additional Supplementary Files [file 41467_2024_55725_MOESM2_ESM.pdf]

Description of Additional Supplementary Files

File Name: Supplementary Data 1
Description: Differential gene expression analysis between low and normal rDNA copy number conditions in GSCs. EA = Expression Analysis data set; CA = Cluster Analysis data set; p_val = unadjusted p value determined by non-parametric Wilcoxon rank sum test; avg_log2FC = log2 fold change expression difference in low rDNA GSCs compared to normal rDNA GSCs; pct_N_rDNA_expressed = percent normal rDNA GSCs gene is express in; pct_L_rDNA_expressed = percent low rDNA GSCs gene is expressed in; p_val_adj = Bonferroni corrected p-value; max.pval = maximum Bonferroni corrected p-value between two analyses (Fig 1D Y acis); plot.FC = minimum effect log2FC (Fig 1D X axis); change = gene up- or down-regulated in both analyses.

File Name: Supplementary Data 2
Description: Spermatogonia gene expression changes of differentially expressed GSC genes. SG Cluster Fold Change = Log2 fold change in low rDNA compared to normal rDNA CN condition in spermatogonia; SG adjusted p-value = Bonferroni corrected p-value from two-sided Wilcoxon Rank Sum Test; GSC Cluster Fold Change = Log2 fold change in low rDNA compared to normal rDNA CN condition in germline stem cells

File Name: Supplementary Data 3
Description: Results of RNAi screen of candidate suppressors of rDNA magnification. RNAi line = RNAi identifier number; BDSC Line Number = RNAi stock line number at BDSC; 95% CI = 95% Confidence interval as determined by standard error of a proportion.

File Name: Supplementary Data 4
Description: List of resources and reagents used in this study. Includes *Drosophila* strains, oligonucleotide sequences, and antibody sources.
