# Supplementary material for: Insulin signaling regulates R2 retrotransposon expression to orchestrate transgenerational rDNA copy number maintenance
Source: Nat Commun. 2025 Jan 4;16:399. doi: 10.1038/s41467-024-55725-6 (PMC11700107; doi:10.1038/s41467-024-55725-6)
Supplement: Supplementary file 7 — Reporting Summary [file 41467_2024_55725_MOESM7_ESM.pdf]

nature portfolio

Corresponding author(s): Jonathan O Nelson, Yukiko M Yamashita

Last updated by author(s): 12-05-24

Reporting Summary

Nature Portfolio wishes to improve the reproducibility of the work that we publish. This form provides structure for consistency and transparency in reporting. For further information on Nature Portfolio policies, see our Editorial Policies and the Editorial Policy Checklist.

Statistics

For all statistical analyses, confirm that the following items are present in the figure legend, table legend, main text, or Methods section.

n/a	Confirmed	
☐	☒	The exact sample size (n) for each experimental group/condition, given as a discrete number and unit of measurement
☐	☒	A statement on whether measurements were taken from distinct samples or whether the same sample was measured repeatedly
☐	☒	The statistical test(s) used AND whether they are one- or two-sided<br>*Only common tests should be described solely by name; describe more complex techniques in the Methods section.*
☐	☒	A description of all covariates tested
☐	☒	A description of any assumptions or corrections, such as tests of normality and adjustment for multiple comparisons
☐	☒	A full description of the statistical parameters including central tendency (e.g. means) or other basic estimates (e.g. regression coefficient) AND variation (e.g. standard deviation) or associated estimates of uncertainty (e.g. confidence intervals)
☐	☒	For null hypothesis testing, the test statistic (e.g. F, t, r) with confidence intervals, effect sizes, degrees of freedom and P value noted<br>*Give P values as exact values whenever suitable.*
☒	☐	For Bayesian analysis, information on the choice of priors and Markov chain Monte Carlo settings
☒	☐	For hierarchical and complex designs, identification of the appropriate level for tests and full reporting of outcomes
☒	☐	Estimates of effect sizes (e.g. Cohen's d, Pearson's r), indicating how they were calculated

Our web collection on statistics for biologists contains articles on many of the points above.

Software and code

Policy information about availability of computer code

Data collection	Cell Ranger (10X Genomics) v7.1.0 used for read alignment of single cell sequencing data. Quantasoft (Bio-Rad) used for ddPCR data collection.
Data analysis	Seurat v4 (Hao et al, Cell 2021) used for analysis of single cell sequencing data. R 4.21. Quantasoft (Bio-Rad) used for ddPCR data analysis.

For manuscripts utilizing custom algorithms or software that are central to the research but not yet described in published literature, software must be made available to editors and reviewers. We strongly encourage code deposition in a community repository (e.g. GitHub). See the Nature Portfolio guidelines for submitting code & software for further information.

Data

Policy information about availability of data

All manuscripts must include a data availability statement. This statement should provide the following information, where applicable:

- Accession codes, unique identifiers, or web links for publicly available datasets
- A description of any restrictions on data availability
- For clinical datasets or third party data, please ensure that the statement adheres to our policy

Sequencing data is available on the NCBI Gene Expression Omnibus. Accession number GSE263351.

Research involving human participants, their data, or biological material

Policy information about studies with human participants or human data. See also policy information about sex, gender (identity/presentation), and sexual orientation and race, ethnicity and racism.

Reporting on sex and gender	NA
Reporting on race, ethnicity, or other socially relevant groupings	NA
Population characteristics	NA
Recruitment	NA
Ethics oversight	NA

Note that full information on the approval of the study protocol must also be provided in the manuscript.

Field-specific reporting

Please select the one below that is the best fit for your research. If you are not sure, read the appropriate sections before making your selection.

- [x] Life sciences
- [ ] Behavioural & social sciences
- [ ] Ecological, evolutionary & environmental sciences

For a reference copy of the document with all sections, see nature.com/documents/nr-reporting-summary-flat.pdf

Life sciences study design

All studies must disclose on these points even when the disclosure is negative.

Sample size	Sample size was not calculated. For all biological samples, the maximum possible sample size was chosen for each type of data.
Data exclusions	Single cell data was excluded after failing quality control metrics. We used standard metrics to identify "cells" within the data set that contained low number or diversity of reads (representing incomplete transcriptomic data from a ruptured cell) or exceptionally high number or diversity of reads (representing multiple cells contained withing the same analysis).
Replication	All data were collected during independent trials on on separate days. All experiments were performed in at least duplicate, and all attempts at replication were successful.
Randomization	Randomization is not relevant to our study, because samples were allocated based on condition or genotype. When possible, genetically identical animals were used within the same condition to control for covariates. Otherwise sibling controls were used for control conditions to control for covariates.
Blinding	Blinding was not done because it was not necessary for our analysis since wild-type control and mutant data are easily identifiable.

Reporting for specific materials, systems and methods

We require information from authors about some types of materials, experimental systems and methods used in many studies. Here, indicate whether each material, system or method listed is relevant to your study. If you are not sure if a list item applies to your research, read the appropriate section before selecting a response.

Materials & experimental systems

n/a	Involved in the study	
☐	☒	Antibodies
☒	☐	Eukaryotic cell lines
☒	☐	Palaeontology and archaeology
☐	☒	Animals and other organisms
☒	☐	Clinical data
☒	☐	Dual use research of concern
☒	☐	Plants

Methods

n/a	Involved in the study	
☒	☐	ChIP-seq
☒	☐	Flow cytometry
☒	☐	MRI-based neuroimaging

Antibodies

Antibodies used	Rat anti-vasa (DSHB AB_760351) used 1:20, Mouse anti-FasIII (DSHB AB_528238) used 1:200, Rabbit anti-pS6 (Romero-Pozuelo et al., 2017, Dev. Cell) used 1:200, Chicken anti GFP (abcam ab13970) used 1:2000.
Validation	Rat anti-vasa has been validated through use over numerous publications, beginning with Aruna et al., 2009. Genetics 181(4) 1437-50. Rabbit anti-pS6 antibody was validated in Romero-Pozuelo et al., 2017 Dev. Cell by genetic hyperactivation and pharmacological inhibition of TORC1 activity and expected increase and elimination of pS6 signal (see Fig. 1A-B, and 1E). Mouse anti-FasIII antibody was generated and validated in Patel et al., 1987 (Cell) in western blots using normal mouse serum as a negative control and immunofluorescence of whole-mount embryos including a deficiency removing the FasIII gene as a negative control (see Fig 6A and 9A-C). Chicken antiGFP was validated by the standards used for abcam commercial production.

Animals and other research organisms

Policy information about studies involving animals; ARRIVE guidelines recommended for reporting animal research, and Sex and Gender in Research

Laboratory animals	Drosophila melanogaster laboratory strains. All stain numbers are provided in table S4. All animals used were 0-5 days old, except for those experiments where ages are specifically indicated, which are 0, 21, and 28 days old.
Wild animals	This study did not involve wild animals.
Reporting on sex	Findings specifically apply to male progeny.
Field-collected samples	There were no field-collected samples in this study
Ethics oversight	Work on Drosophila melanogaster does not require ethical oversight or experimental approval.

Note that full information on the approval of the study protocol must also be provided in the manuscript.

Plants

Seed stocks	NA
Novel plant genotypes	NA
Authentication	NA
